# Clinical evaluation of patients with moderate to severe Alzheimer disease

**DOI:** 10.1590/S1980-57642008DN10300012

**Published:** 2007

**Authors:** Paulo Rogério Borges Rosmaninho Varandas, Rossana Russo Funari

**Affiliations:** 1 Serviço de Geriatria do Hospital das Clínicas da Faculdade de Medicina da Universidade de São Paulo.

**Keywords:** Alzheimer disease, therapy, comorbidities, aged

## Abstract

Today, Alzheimer disease has become a serious risk to individual and public health, due to the significant incapacity it causes patients, its influence on family members and caregivers, along with the ensuing direct and indirect costs.

**Objectives:**

To build the profile of patients with moderate/severe AD, in the Geriatric Clinic Service of Cognitive Alterations of the Medical School at Universidade de São Paulo, by studying demential and comorbidity conditions and the degree of effectiveness of the therapies applied.

**Methods:**

30 patients with moderate or severe AD were selected, (77.8±7.29 years). Age, sex, schooling, prevalent comorbidities/treatments and respective clinical-laboratorial effectiveness were analyzed. Instruments were applied to evaluate the cognitive and behavioral condition and dementia control therapies.

**Results:**

Most frequent comorbidities were arterial hypertension (80%) and diabetes (43.3%). A maximum dose of rivastigmine was observed in 43% of the patients, where 76% experienced adverse effects. Severe patients presented more cases of uncontrolled comorbidities, such as hypertension (P<0.001), as well as more behavioral alterations (P<0.001) and functional loss (P=0.004). Patients with greater behavioral alterations proved to be more functionally dependent (P=0.002), having less comorbidity control (P=0.004).

**Conclusions:**

In this population, a high incidence of comorbidities, frequent behavioral alterations and difficulties in therapy management were noted due to the severity of the dementia condition. New therapies for more adequate control of severe dementia should be studied.

## Abstract (pt)

Hoje em dia, a doença de Alzheimer (DA) tornou-se um sério risco de saúde individual e coletivo, em decorrência da significativa incapacidade que acarreta aos pacientes, das influências sobre os familiares e cuidadores, além dos custos diretos e indiretos que ocasiona.

**Objetivos:**

Traçar perfil dos pacientes portadores de DA moderada/grave, do Ambulatório de Alterações Cognitivas – Serviço de Geriatria da Faculdade de Medicina da Universidade de São Paulo, estudando-se quadro demencial, comorbidades e grau de eficácia das terapêuticas aplicadas.

**Métodos:**

Selecionados 30 pacientes com DA moderada e grave, (77,8±7,29 anos). Analisou-se idade, sexo, escolaridade, comorbidades prevalentes/tratamentos e respectiva eficácia clínico-laboratorial. Aplicaram-se instrumentos específicos para avaliação do quadro cognitivo,comportamental e avaliação da terapêutica para controle demencial.

**Resultados:**

Comorbidades mais freqüentes: hipertensão arterial 80% e diabetes 43,3%. Uso de rivastigmina em dose máxima ocorreu em 43% pacientes, 76% efeitos colaterais. Pacientes graves apresentaram mais casos de comorbidades descompensadas, como hipertensão (P<0,001), mais alterações de comportamento (P<0,001) e perdas funcionais (P=0,004). Pacientes com maiores alterações comportamentais apresentaram-se mais dependentes funcionalmente (P=0,002) e com pior controle de comorbidades (P=0,004).

**Conclusões:**

Nessa população estudada observou-se alta incidência de comorbidades, alterações comportamentais freqüentes, dificuldades no manejo terapêutico, decorrentes da gravidade do quadro demencial. Novas terapêuticas devem ser pesquisadas para controle mais adequado de demenciados graves.

Today, AD has become a serious risk to individual and public health, due to the significant incapacity it causes patients, its influence on family members and caregivers, along with the ensuing direct and indirect costs. It is known that, considering the substantial increase in the aged population, there has been a proportional increase in degenerative diseases, such as AD.^1,2^ In Brazil, for example, a recent study undertaken in the urban area of Catanduva-SP, AD was responsible for 55.1% of dementia cases. Prevalence increases with age, varying from 0.16% among patients aged between 65 and 69 years, to 23.4% in those equal to or older than 85 years. The international tendency is toward a progressive increase in the prevalence rates, being more accentuated in developing countries such as Brazil, due to the unbridled ageing of the population.^4^

AD develops slowly and progressively, generally evolving over many years, compromising superior cortical functions, principally temporal and parietal, leading to disturbances in memory, language, executive functions and visuo-spatial abilities.^1,3^ Those patients in a moderate phase present significant difficulties in daily-life, such as loss of personal hygiene, non-recognition of objects and closely-related people, neuro-psychiatric symptoms, among others. Evolving to the severe stage, the worsening of disturbances in activities basic and instrumental to these patients, frequently results in the need for institutionalization. These patients can evolve to generalized rigidity, walking difficulties and apathy, being bedridden and the need for a feeding tube, increasing the risks for pulmonary and urinary infections.^1,3,4^ The evolution, diagnosis, and treatment for Alzheimer dementia have been the subject of many recent studies, resulting in daily advances in scientific knowledge. However, little attention has been given in the literature to the existent comorbidities in patients with dementia.^5^ These patients are often excluded from research, or suffer from outdated concepts such as the belief that demented patients have a lesser number of chronic illnesses and therefore are “healthier” than the general public. In reality, these patients present a greater number of comorbidities, with greater cognitive compromise, greater annual cost and, principally, higher mortality rates.^5-7^. The objectives of this study were to build a profile of moderate and severe Alzheimer patients, evaluating cognition, alterations in memory and behavior, through interviews with patients and caregivers together with the application of tests to analyze these factors and cite the most common clinical comorbidities.

This study was based on the reality of these patients with moderate or severe Alzheimer disease, and mixed vascular-Alzheimer dementia.

## Methods

The research was based upon the analysis, through a transversal observational study, of patients with moderate to severe Alzheimer disease or mixed dementia, through previously scheduled interviews and with the consent of those legally responsible for the patients. The sample was comprised of 30 patients, set in accordance with the order of entry to the study, independent of sex and drawn from the Cognitive Geriatric Outpatient Group (CGOG) – Geriatric Discipline of Internal Medicine Division- Clinicas Hospital School of Medical Sciences. Approximately one-fifth of the entire body of catalogued patients in the cited outpatient services was analyzed. The interviews took place between April and December of 2005. The inclusion criteria were the following: patients with a minimum of sixty years of age, selected by dementia severity as measured by the Mini-Mental State Examination (MMSE)^9^ – scoring between three and fourteen points, and using the Clinical Dementia Rating (CDR)^10^ – with scores greater than or equal to two points, all taking a therapeutic dose of rivastigmine (greater than or equal to six mg/day). The exclusion criteria were: patients aged less than sixty years, not diagnosed with Alzheimer disease or mixed dementia, initial phase of dementia as defined by the CDR and MMSE criteria, not taking rivastigmine or a dosage less than six mg/day, and those that did not wish to enroll on the protocol. The patients and/or those responsible for them, signed a Free and informed Consent Term, according to the standards set forth by the Ethics Committee of Clinicas Hospital.

The patients were identified by name, gender and level of schooling; presented in a clinical file, noting the most prevalent comorbidities/associated treatments and the respective degree of effectiveness evaluating clinical and laboratorial aspects.

The clinical data was obtained during the interview, through anamnesis, physical examination, evaluation of the compendium and medical prescriptions and laboratorial exams. The patients/caregivers were questioned about the medication therapy for dementia control – rivastigmine – where dosage taken, tolerance, effectiveness and principal adverse effects were noted, as well as frequency of cholinesterase inhibitors, anti-depressives and anti-psychotics and their consequences.^11^

Specific tests were applied to the patients to evaluate dementia state and provide a cognitive analysis.

The first group of tests administered was the MEEM. In this study, a score of three to fourteen points was considered indicative of moderate to severe dementia. Two measures were considered in measuring cognitive function, verbal and performance scores, low scores may have been related to other conditions, such as depression and delirium.^9,10^

Other variables studied were functionality evaluation tests, such as evaluation of basic daily activities (EBDA),^11^ investigating whether or not the patient was able to perform tasks without assistance, such as bathing, dressing, going to the bathroom, moving, feeding themselves and continence control. The evaluation of instrumental daily activities (EIDA),^11^ analyzed the patient’s capacity to use the telephone, perform shopping, planning, preparing and serving of food, helping in domestic chores, washing clothing, using public transportation, understanding the handling of money, and administering medication. The scale of Daily Living of the Alzheimer Disease Co-operative Study modified for severe illness (ADCS-ADLsev)^11,12^ was devised to evaluate performance, which ranges from the highest level to complete function loss. This is based upon interviews with caregivers, focusing on the most common and consistent performance of the daily living activities during the last four weeks. In this study, the subgroup of nineteen items was chosen to evaluate the functional capacity of the sample population to eat, walk, go to the bathroom alone, perform personal hygiene, comb one’s hair, dress oneself, use the telephone, communicate, clear the table after a meal, find one’s belongings, drink, discard garbage, leave the home, stay alone, close off the tap, and turn lights on and off. A total score of fifty-four points represented an excellent score, while a lower score indicated a lesser performance.^12^ The global scales reflected the approach in a common clinical situation, serving for diagnosis, while rating and monitoring response to the treatment. The CDR,^10^ considered to be the gold standard in classification of dementia severity, was used in this study to evaluate the influence of cognitive loss on the ability to conduct daily activities. Six categories were evaluated: memory, judgment, temporal-spatial orientation, problem solving, social relationships, pastimes and personal care. Each category had separate classification, and through this a final classification was obtained: 0 (normal), 0.5 (questionable), 1(slight), 2 (moderate) and 3 (severe). The scale was applied exclusively with the caregivers.

The Neuropsychiatric Inventory (NPI)^13^ was the scale selected to evaluate the behavior of the patients in this study. The NPI score included a series of twelve items including hallucinations, agitation, aggressiveness, anxiety, euphoria, apathy, indifference, lack of inhibitions, irritability, aberrant motor behavior. This included a severity score and scores for the frequency of each item, where the result was then calculated by multiplying the total severity score by the total frequency score. The NPI total was the sum of the total scores of each subscale and the frequency value, from zero – absent, to three – very intense. The NPI also included a subjective evaluation of the caregiver’s responsibility for each subscale, on a scale from one to five. Evaluating the patient, the scoring went from zero to one-hundred-four and from zero to sixty to evaluate the discomfort of the caregiver, with zero indicating the ideal for each case.^13^

## Results

Of the one-hundred-fifty patients catalogued at the CGOG, thirty patients were selected between the months of April and December of 2005. There was no discontinuance, refusals, deaths or violations of the study. Of the patients analyzed, 56% were female, with a median age of 77.8±7.29 years, and a median schooling of 2.92±2.62 years. Of all of the evaluated comorbidities, the most prevalent was systemic arterial hypertension (SAH), comprising 80% of the sample population. Of those identified as hypertensive during the interview, 75% presented blood pressure values above the level recommended in the literature as the treatment goal (arterial pressure <140x80 mmHg).^6^ Only 15% of the uncontrolled hypertensives used monotherapy, not recommended in the literature,^6^ and 68% were sedentary, without any type of diet restriction. Another prevalent comorbidity was Diabetes Mellitus (DM), affecting 43.3% of the sample population, with 47% presenting fast blood sugar with on-site values greater than 110 mg/dl. According to the caregivers, 52.3% of the diabetic patients were on adequate diets, with 31% of caregivers receiving orientation from a nutritionist. Depression was observed in 40% of the patients, with 75% of patients uncontrolled by the DSM-IV standard. A total 80% of the depressive patients received anti-depressive medication, most often at the maximum dosages or in evolution. Table 1 lists all of the comorbidities present in the sample population.

**Table 1:** Occurrence of each comorbidity studied in the sample population.

	No	Yes		No	Yes
			Chronic		
			Obstructive		
Anemia	26 86.7%	4 13.3%	Pulmonary Disease	29 96.7%	1 3.3%
Systemic arterial hypertension	6 20.0%	24 80.0%	Vascular insufficiency	19 63.3%	11 36.7%
Diabetes	17 56.7%	13 43.3%	Urinary incontinence	21 70.0%	9 30.0%
Depression	18 60.0%	12 40.0%	Stroke	27 90.0%	3 10.0%
Osteoarthritis	21	9	Glaucoma	28	2
	70.0%	30.0%		93.3%	6.7%
Osteoporosis	23 76.7%	7 23.3%	Cataract	25 83.3%	5 16.7%
Benign prostate hypertrophia	24 16.7%	6 20.0%	Hypothyroidism	28 93.3%	2 6.7%
Hypothyroidism	28 93.3%	2 6.7%	Dyslipidemia	18 60.0%	12 40.0%
Parkinson disease	27	3	Uterus cancer	29	1
	90.0%	10.0%		96.7%	3.3%
Cardiac insufficiency	29 96.7%	1 3.3%	Breast cancer	29 96.7%	1 3.3%
Coronariopathy	26 86.7%	4 13.3%	Renal insufficiency	29 96.7%	1 3.3%
Arrhythmia	29 96.7%	1 3.3%			

Control of dementia with the use of rivastigmine, in progressive dosages up to the maximum doses of the medication (12 mg daily), which was well-tolerated, although not without adverse effects. Some 43% of the patients were taking maximum doses of the medication, with an adherence rate of 66% with 76% having adverse effects at some point during the therapy. The principal adverse effects with the use of the medication were weakness (43%), feeling ill (36%), loss of appetite (23%) and headache (16%). Nonetheless, most of these effects were of a slight to moderate degree, and suppressed by adjusting the medication dosage. The use of neuroleptics was prevalent, with 33% of the sample population taking risperidone, periciazine (20%) or quetiapine (16%) at maximum doses. A total of 73% of caregivers reported an improvement in the patients upon using these drugs, effectively controlling insomnia and agitation, while 68% reported increasing these drugs on their own, without medical orientation.

**Figure 1:**
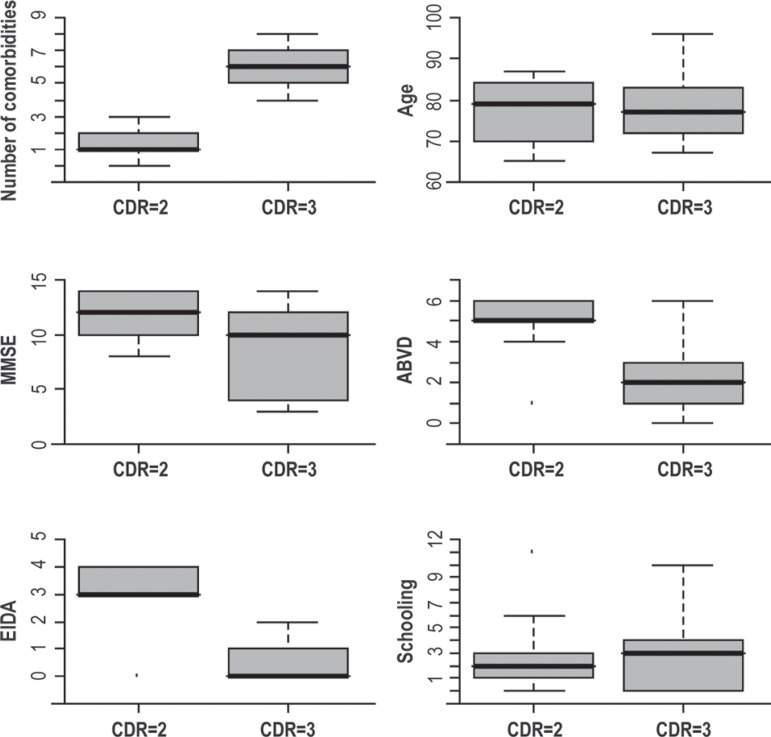
Relationship of CDR with all other evaluation scales of the dementia condition.

**Figure 2:**
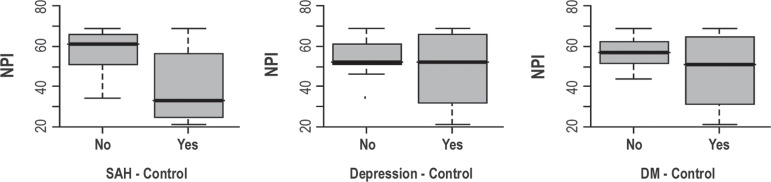
Relationship between NPI and SAH/MD control, observing relationship between behavior alterations and more prevalent comorbidity control.

**Figure 3:**
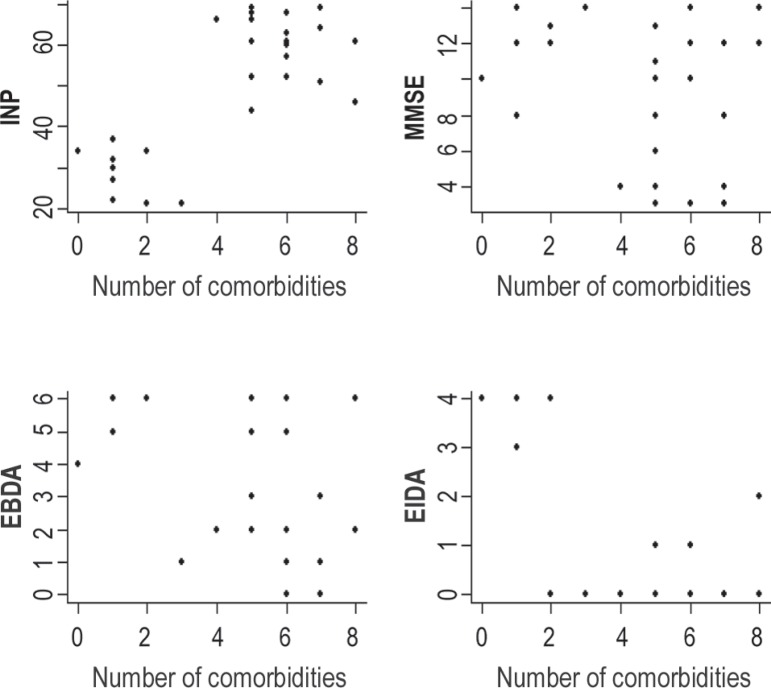
Relationship among comorbidities and dementia evaluation scales.

The mean score of the sample population on the MMSE was 9.56±3.72, with compromise most accentuated in attention and calculations (simple subtraction operations) and memory fixation (remembering previously repeated words), with 76% of patients obtaining a score of zero on these questions. The median score on the EBDA scale was 3.23±2.14, with greater loss in control of incontinence and loss of personal hygiene. The mean score on the EIDA scale was 1.03±1.5, with relevant loss in the handling of money and use of means of transportation. On the ADCS-ADLsev scale there was a mean score of 27.8±13.12, with principal loss in leaving the house, talking, turning lights on/off. On the NPI scale, a median score of 50.26±16.17was observed, with more relevant scores on questions such as agitation, anxiety and apathy. It is worth emphasizing that we encountered higher NPI scores than those found in the literature.^3,6,13^ Of the patients analyzed, 30% were standard for moderate dementia, according to the CDR scale (CDR=2). Through the scoring analysis of different scales, calculating scores-resume, and the Student t-test, it was evident that the more severe patients (CDR=3) had demonstrated greater behavior alterations – higher NPI scores (p<0.001), were more functionally compromised – higher EBDA/EIDA (p=0.004), and had a greater number of comorbidities (p<0.001), as Figure 1 demonstrates. The more severe patients presented higher MMSE scores (p=0.003) and lower ADCS-ADL severity-scores (p=0.001). There was no relationship between dementia severity and schooling (p=0.639). The association between comorbidity control and CDR was demonstrated in the application of the Fisher exact-test. An association between CDR and SAH controls (descriptive level=0.001) and DM (descriptive level=0.011) was detected. The patients with more behavior alterations (higher NPI) also showed a relationship with higher MMSE rates (p<0.001), ABVD (p=0.002), age (p<0.001), schooling (p=0.004); as well as SAH control (p=0.004) and DM (p=0.03). This relationship was verified through observation of scores-resume, box plots, and the Student t-test for non-related samples, and can be seen in Figure 2. In order to study the association between the number of comorbidities and NPI, MMSE, EBDA and EIDA, dispersion graphs were created (Figure 3) and Pearson linear correlation coefficients calculated (Table 2). A strong association was verified between number of comorbidities and the NPI and EIDA variables. The association with EBDA can be classified as moderate. The association between comorbidity control and the scales (the Student t-test) for the sample population and the scores-resume indicated a relationship between, as previously mentioned, NPI and DM and SAH control, as well as between EIDA and SAH control (p=0.028)

**Table 2:** Pearson coefficient - linear correlation among the variable number of comorbidities and Neuropsychiatric Inventory (NPI), Mini-Mental State Examination (MMSE), Basic Activities of Daily-living (BADL) and Instrumental Activities of Daily-living (IADL).

	Coefficient			Confidence intervals	
Variables	NPI	0.742		0.520	0.869
Number of comorbidities	MMSE	-0.225		-0.541	0.146
	BADL	-0.516		-0.738	-0.191
	IADL	-0.751		-0.874	-0.536

## Discussion

In the population studied a high prevalence of comorbidities, behavioral alterations, and therapy management difficulties were observed.

Alzheimer patients can present a range of cognitive disturbances, in the most diverse areas, such as memory, behavior, and functional dependence and can expect varying responses. This diversity is even further intensified when the target of the study is patients with greater severity.^4^ This cognitive loss presents a multifactorial etiology and depends not only on the natural evolution of the disease, but also on the clinical comorbidities presented by the patient. These may include sensorial disturbances, adverse effects of medication, environmental factors, caregiver variations, as well as psychological and behavioral states.^14-16^ A greater number of uncontrolled diseases was seen (compared to the literature) and their relationship to severe cognitive decline was observed. In spite of adequate pharmacological intervention, diseases such as SAH, diabetes and depression manifested high rates of decompensation. Frequently, poor adherence to the treatment, absence of non-pharmacological therapies (high sedentary rates, absence of diet controls, among others), delayed diagnosis, sensorial disturbances, little attention to possible infections, nutritional disturbances, poor control of non-related symptoms such as pain (patients with severe cognitive compromise, displaying important disturbances in language, expression, comprehension, etc) and even falls/fractures can all contribute to clinical decompensation and decline in cognitive control.^17^ Patients taking many medications, their greater adherence to the use of more drugs and the consequent greater incidence of adverse effects can all contribute to clinical decline.^16,17^ This occurs not only because of the use of the drugs oriented to control dementia and its comorbidities, but also because of the indiscriminate use of neuroleptics, “tranquilizers”, benzodiazepines that, having a long shelf-life, caregivers view as the solution to diminish their own anxiety, due to the desire to revert the patient’s agitation. Often, simpler conduct, or adequate medical evaluation are able to revert the condition without resorting to indiscriminate doses of drugs which carry a high probability of adverse effects.^15,16^

In our study, higher prevalence of behavioral disturbances was observed compared to the literature.^3,6,13^ Patients with more behavioral disturbances had a greater propensity for a higher number of comorbidities, more clinical decompensation, greater use of medications and their adverse effects, as well as a greater dementia severity on the CDR scale. Clinical control becomes more difficult, possibly, due to the greater number of factors interfering in the case, with a worsening of cognition and a greater propensity for clinical decompensation. We should also consider that given the difficulty for the aged to adequately control the diverse comorbidities/treatments and maintain life-quality, then it stands to reason that in severely demented patient this becomes even more challenging. This could occur because of the difficulty in reporting symptoms and adverse effects, due to the limitations imposed by the cognitive disturbance, along with a lesser capacity to decide, difficulty in adhering to treatment recommendations, together with the greater risks of diagnostic and therapeutic procedures.^4,5,7^

The study group displayed a significant compromise in the capacity to independently perform daily operational and basic activities, such as personal hygiene, communicating adequately, turning lights on/off, demonstrating disturbances in various cognitive aspects-such as the impossibility of memorizing simple things and calculating. All of these compromises complicate the patients’ life quality, and frequently make them totally dependent on their caregivers. The scales focus on the most-frequently encountered alterations in AD, which does not necessarily mean that they are present or are the most important to all the patients. In accompanying the cases, the objectives need to be individualized, selecting the most relevant alterations, for example, behavioral, observing these factors with greater attention when evaluating the effectiveness of the therapy.^18^ Due to the high rates of behavioral alterations found in this study, we can verify that rivastigmine, neuroleptic drugs, the dosages administered and other therapeutic actions are not capable of adequately controlling behavioral alterations. Non-pharmacological measures (such as diet, environmental concerns-avoiding stressful factors, avoiding sedentary lifestyle, psychological support), adequate adherence to medication, responsible use of medication with high risk of adverse effects, adequate control of symptoms and comorbidities are some of the measures that should be encouraged and recommended to the caregivers.^15,16^ Furthermore, it is important to stimulate the patient’s intact skills so as to increase their autonomy.

The search for optimal comorbidity treatment, with earlier diagnosis, control of dementia and its symptoms should always be the objective of the medical team. Often, simple interventions can have important effects on the symptoms and functionality while the risk-benefit of all conduct should always be questioned, ensuring they are not absent or excessively aggressive. The family is the fundamental element in accompanying these patients, as well as representing a precious source of information, and should always participate in decisions and interventions.^5^

In the population studied a high incidence of comorbidities, frequent behavioral alterations, and therapy management difficulties were observed, where these were of a multi-factorial nature including those due to dementia severity. In this sample, a higher number of elements and scales for the more severely demented patients facilitated an improved evaluation of the condition. New therapies for a more adequate control of severe dementia should be studied.
